# Perceived factors influencing the initiation of methamphetamine use among Akha and Lahu youths: a qualitative approach

**DOI:** 10.1186/s12889-019-7226-y

**Published:** 2019-06-28

**Authors:** Chalitar Chomchoei, Tawatchai Apidechkul, Pilasinee Wongnuch, Ratipark Tamornpark, Panupong Upala, Marisa Poomiphak Na Nongkhai

**Affiliations:** 1 Center of Excellence for The Hill tribe Health Research, Mae Fah Luang University, Chiang Rai, Thailand; 2 School of Health Science, Mae Fah Luang University, Chiang Rai, Thailand

**Keywords:** Akha, Lahu, Hill tribe, Youths, Initiation, Methamphetamine

## Abstract

**Background:**

Methamphetamine use not only impacts health and the economy but also causes social impairment, particularly among the poorly educated and underprivileged young populations among the hill tribes in northern Thailand. Youths are the most vulnerable population for methamphetamine use due to various factors, including parenting styles, childhood exposure, and location of the village. This qualitative approach aimed to investigate the perceived factors influencing the initiation of methamphetamine use among the Akha and Lahu youths in northern Thailand.

**Methods:**

A qualitative approach was used to elicit the information from key informants of Akha and Lahu youths who lived in villages in Chiang Rai province. A sixteen-question guideline was developed and examined for quality by three experts in the field and piloted before use. In-depth interviews were conducted among 19 Akha and 22 Lahu youths, serving as key informants from four villages, in a private and confidential room in their villages between June and August 2018. Each interview lasted approximately an hour. A thematic analysis was performed to evaluate the information.

**Results:**

In total, 41 participants (19 Akha and 22 Lahu) from 4 villages participated in the study. According to the context and content obtained, four major perceived factors (low self-esteem, family member use, positive expectation regarding methamphetamine use, and availability), and five supportive factors (social norm perception, school dropout, family level problems, poor economic, and no Thai citizenship) were found to contribute to the initiation of methamphetamine use among the Akha and Lahu youths in northern Thailand.

**Conclusions:**

Akha and Lahu youths are initiating methamphetamine use due to several factors, including living in a poor family and in a remote area. All relevant government agencies with a mission to prevent and protect against methamphetamine use should consider the perceived factors influencing the initiation of methamphetamine use in these populations in order to develop a powerful program to stop methamphetamine use.

**Electronic supplementary material:**

The online version of this article (10.1186/s12889-019-7226-y) contains supplementary material, which is available to authorized users.

## Background

Today, Thailand has been classified as one of the countries facing a problem of illegal drugs, particularly methamphetamine, which is the 1st ranked of substance used among the youths in 2018 [1]. The Office of Narcotic Control Board Ministry of Justice reported that the number of people who were arrested for methamphetamine use and sale increased every year, particularly in the northern region of Thailand, with 19,557 arrested cases (70/10,000 population) in 2017 [2]. The northern region, particularly in the border areas of Thai-Myanmar and Thailand-Laos, are locations of methamphetamine production [2]. In 2017, more than 27 million tablets of methamphetamine were confiscated [2]. The Lahu (23%) and Akha (11%) tribes were identified as methamphetamine agencies which were higher proportions than groups of people in Thailand [2].

Methamphetamine use impacts an individual’s physical, economic, social and mental health [3]. Brain impairment is a major health impact among individuals who use methamphetamine, particularly among early users [4]. People aged 15–24 years, considered youths under the United Nation Definition [5], are the most vulnerable population for engaging in methamphetamine use, particularly among those who are living in rural and poor settings [6]. There are several reasons prompting the initial use of methamphetamine [7]. Additionally, there might be different reasons for initiation of methamphetamine use in different age groups [8]. Different locations of living have different factors that contribute to the use of methamphetamine among the defined vulnerable population [9]. Hill tribes are considered to be one of the vulnerable populations for methamphetamine use in Thailand, particularly among those aged 15–24 years old [5, 9].

Youths are the stage of the transition from dependence to adulthood [10]. With a feeling of independence at this stage, these youths might be exposed to both desirable and undesirable experiences. Some youths are able to handle it well; however, others have already begun using drugs, such as methamphetamine and marijuana. Many conditions and factors might motivate youths to engage in the use of drugs, especially methamphetamine. Many studies have reported that parenting style, personal perception and life expectations, family’s economic status and community environment are motivating factors for methamphetamine use among youths [11].

A hill tribe is a group of people who have migrated from South China after the nineteenth century and settled along the mountainous zones in northern Thailand. These tribes are classified into six main groups, namely, the Akha, Lahu, Karen, Hmong, Yao, and Lisu, with each having its own unique culture, language, and lifestyle [12]. The Akha and Lau are the largest and second-largest groups, respectively, of the hill tribes in Thailand [13]. The tribes’ villages are located in very remote areas, particularly at the borders of Thailand-Myanmar and the Republic of Laos [13]. With the remote location of the villages, poor living conditions, and low economic and educational status, the hill tribes are prone to being drug agents. Ultimately, the tribes have become a marginalized population with a triple stigma: poor, hill tribe members, and drug agents [14].

A large proportion of the Akha and Lahu people are living under poor economic and educational conditions and plant economically unviable crops to help their families survive [13]. Due to economic constraints, these farmers spend most of their day on the farm while leaving their children in the village. Many Akha and Lahu children have different exposures during their childhood. The patterns of drug use have not been previously documented among the Akha and Lahu youths in these areas. However, a study reported a pattern of methamphetamine use among Thai youths that began with being persuaded by their peers and evolved into being users in later years [15]. Ultimately, some youths decided to initiate methamphetamine use, while others did not. There are many motivating factors for the initiation of methamphetamine use, such as parenting styles and family relationships during childhood. However, there is no scientific information available regarding the initiation of methamphetamine use among Akha and Lahu youths in Thailand. This study aimed to investigate all perceived factors influencing the initiation amphetamine use among Akha and Lahu youths who are living in Chiang Rai Province, Thailand.

## Methods

### Study design and tools

A qualitative approach was used to elicit information from selected key informants on all perceived factors influencing the initiation of methamphetamine use by researchers who graduated in the field of qualitative research and had years of relevant research experience. Participants were interviewed to get information on their perceived reasons for initiation amphetamine use, and about their family, life context, etc. which related to initiate the methamphetamine use.

Key informants were Akha and Lahu youths aged 15–24 years with experience using methamphetamine at least once in their life before the date of the interview.

A sixteen-question guideline (Additional file 1) was developed from the literature and from prior interviews with Akha and Lahu community leaders. Example questions are as follows: “What do you think about methamphetamine? Does it impact your life? Your family? Your friends? Your village?” “What are the major factors that they contribute to your decision in initiation use methamphetamine?” The questions were developed by the researchers. The purpose of this stage was to ensure that the intended content and context were covered and that the sixteen-question guideline targeted the essential details. The questions investigated such topics as personal perception of methamphetamine use, parenting styles, childhood experience of exposure to methamphetamine, family relationships, life perspective, and perception of the use of methamphetamine.

The sixteen-question guideline was examined for quality by three external experts in the relevant field. The guideline was also piloted for reliability and feasibility from February 2–8, 2018, among two Akha youths (male and female) and two Lahu youths (male and female) who lived in the Akha-Santisuk village and the Lahu-Cha-Lae village in Mae Chan District in Chiang Rai Province. After improving the quality of the question guideline through the pilot test, it was ready to use in the field.

### Study setting and study population

The study was conducted in two selected Akha villages, namely, Arku village (small and close to the city) and Hua Eurn village (large and remote village), and in two selected Lahu villages, namely, Hua Purng village (large and close to the city) and Hua Mau village (small and remote village). All of these villages were located in the Mae Fah Laung District in Chiang Rai Province. The study participants were Akha and Lahu youths aged 15–24 years who were living in the selected villages.

The selection criteria were a) being Ahka or Lahu, b) being aged 15–24 years, and c) having experience using methamphetamine at least once before the interview date. However, those who were unable to speak Thai and unable to identify themselves as either Akha or Lahu were excluded from the study.

### Data gathering procedures

Permission to approach the villages and participants was granted by the district government officers. Afterward, we contacted the headmen from the Akha and Lahu villages to ask for their cooperation in our research in their villages. The first individual who used methamphetamine and met the inclusion and exclusion criteria was introduced by the village headman. Participants were selected by a snowball technique. The next 3–5 persons who used methamphetamine were introduced by the first participants or snow-ball techniques. Then, the selected persons were contacted and asked for their participation in the study via phone call before an appointment was made for an interview. The interview approach was gender-matched, which means that a male interviewer interviewed male participants and a female interviewer interviewed female participants. After the voluntary consent form was obtained from the selected participants, the interviews were conducted in a private and independent room between June and August 2018. Before asking questions, interviewers established rapport with interviewees through different techniques, such as asking questions and making eye contact, until they had a positive relationship with the interviewees. Interviewers expressed their goal and research process, including the need for cooperation from participants, before starting the interview. A face-to face method was used for the interview. The interviews were conducted at each participant’s home in a village and lasted for an hour each. Interviews were taped and noted upon agreement with the participants. All selected participants were invited for interviews more than once until the data were saturated (12 interviewees, 4 Akha and 8 Lahu).

### Data analysis

All taped interviews were typed into transcriptions by researchers and checked for errors. Transcripts were returned to a participant for comment and correction. There were 246 pages of transcripts from 41 stories/participants. All transcripts were reviewed again before analysis and extraction into their thematic form. All transcripts and field notes were entered into the NVivo program and were coded line-byline based on the key issues, ideas, and perceptions of the initiation of methamphetamine use among the participants. A thematic analysis was used to construct the results from the interviews with the NVivo program (NviVo, qualitative data analysis software; QSR International Pty Ltd. Version 11, 2015). There were 36 codes or key issues obtained from the program. All codes were grouped into major and supportive factors related to the initiation of methamphetamine use among the Akha and Lahu youths. Afterward, the results were revised and critiqued by the research team before a conclusion was developed.

## Results

There were several patterns of substance use among the Akha and Lahu youths. Most males started with alcohol use after age 10, followed by smoking. Alcohol use among the Akha and Lahu men was related to their religious rituals. With access to low-priced alcohol and with the ability to buy alcohol in the small village grocery, Akha and Lahu youths use alcohol at a young age (younger than 12 years, on average). Beer was the youths’ favorite and was often used by all members of the peer groups. There was no difference in the use of alcohol by sex or tribe among the youths. Regarding the pattern of smoking, a greater proportion of males smoked than females, particularly among the Akha participants. Smoking was not common in either Akha or Lahu female youths. The other major substance used among the Akha and Lahu youths was methamphetamine. There was no other substance in use among the Akha and Lahu youths.

In the analysis, two groups were classified according to the context and content obtained from the participants; major and supportive factors. The classification was also made based on the impact of a factor to contribute to the initiation of methamphetamine use including the information obtained from the specific question of “what are the major factors that they contribute to your decision in initiation use methamphetamine?”. Finally, four major perceived factors and five supportive factors were found to contribute to the initiation of methamphetamine use among the Akha and Lahu youths in northern Thailand.

### Four major perceived factors

Major perceived factors were defined as main or direct factors that affected the initiation of methamphetamine use among the Akha and Lahu youths. Four factors were extracted from the analysis.

#### Low self-esteem

Low self-esteem was the first perceived factors influencing the initiation of methamphetamine use among the Akha and Lalu youths of both sexes. Individuals who used amphetamine of both sexes and in both tribes indicated that they had low self-esteem. They could not identify their strong points themselves. People who have low self-esteem act based on others’ ideas or commands, particularly those of their parents. The majority reported that they were following the commands or orders of their parents regarding what to do or not to do in everyday life. Then, those with the characteristic of having low self-esteem were more easily motivated to use methamphetamine by their peers.

A 17-year-old Akha man said the following:

*“I think I have nothing good compared to others. I always listen to my father for what to do every morning. My mother also told me that a good son should follow his parents. Yes, one day last year, I decided to use the drug (methamphetamine). I bought a pill for 50 baht from one of my friends and started to use it. There are 3 different prices of pills: 50, 100, and 130 baht per pill. Different prices will determine the levels and strength of the drug.”* (Akha male youth, 17 years old, # 02).

A 15-year-old Lahu woman said the following:

*“I failed exams many times while I was studying in school. My parents told me that I could not be a good woman anymore. Every girl in my village listens to their parents. Those who did not listen to their parents have moved out of the village for work, but I did not. I think that is not correct, and I cannot also do that. I have thought a lot on my life and decided to use the drug (methamphetamine) eventually. While I use it, I feel happy and nobody can force me.”* (Lahu female youth, 15 years old, # 21).

However, two Lahu men and four Akha men had great self-esteem. Some of them believed that they were the only ones who could support their families financially; without them, their families would be terrible.

#### Using methamphetamine in the family

A large proportion of individuals who used amphetamine reported that at least one of their family members used methamphetamine while they were little boys or little girls. Twenty-nine people reported that at least one of their parents used methamphetamine. Many people reported having a family member who used methamphetamine and that they became involved in the use of methamphetamine through their family member. Among those who were exposed to a family member who used methamphetamines, their decision to initiate methamphetamine use was highly likely, particularly among those who had an additional risk factor, such as living in a poor family or having parents with poor education. Youths who had a nonnegative perception of drugs or methamphetamine use while they were young tended to start using methamphetamine in later years.

A 19-year-old Akha man said the following:

*“I have known about drug (methamphetamine) use from my father. One day a few years ago, he asked me to use it. I am very familiar with how to use and where to buy it.”* (Akha male youth, 19 years old, # 11).

A 24-year-old Lahu woman explained the following:

*“It is ok for me to use the drug (methamphetamine) because at least three of my relatives use it. Do you know, if you want to work effectively, you need one or two pills a day, and then you can work a whole day without feeling weak.”* (Lahu female youth, 24 years old, # 22).

An 18-year-old Akha woman said the following:

*“I had a grandfather and grandmother who used opium before passing away two years ago. They told me that opium or the drug (methamphetamine) was good for one’s health and for improved energy for daily work.”* (Akha female youth, 18 years old, #18).

She added the following:

“*I started using methamphetamine last year, January 2017, during a new year’s party with friends. I do not have any negative perception of my initial use of the drug.”* (Akha female youth, 18 years old, #18).

#### Positive expectation of methamphetamine use

A large proportion of Akha and Lahu youths have positive expectations of methamphetamine use, particularly during their first or initiation use. A positive expectation of methamphetamine use plays a crucial role in the initiation of methamphetamine use in Akha and Lahu youths.

An 18-year-old Lahu woman said the following:

*“I feel happy while using it. Do you know I am facing many problems in my life and in my family? I think it is good to use; it makes me feel happy.”* (Lahu female youth, 18 years old, #18).

A 23-year-old Lahu man expressed the following:

*“Many people in my village talk positively about the drug (methamphetamine) and the good points. They usually tell me that it is good to use because while using it, you can work more and then you can earn more money. Today, earning money to support everyone in a family is very difficult, right?! Sometimes, we do not have money for medicine when some of our family members become ill. Sometimes, we do not have cash to buy food for family members. Many people in my village said that using the drug helps them make more money and be happy!”* (Lahu male youth, 23 years old, #13).

A 16-year-old Akha man said the following:

*“I have used the drug (methamphetamine) for 3 years, and I have been asked to help police spy on drug agents in this village. Yes, I helped the police, but I had to move to live in Chiang Mai for two years to avoid the wrath of drug agents. I just came back to the village for 3 weeks. I personally think that it is not a good thing to use the drug but I need to use it for work. If I did not use it, I would have no energy to work on the farm, and everyone uses it, even adults in my village.”* (Akha male youth, 16 years old, # 08).

#### Availability

Remote villages have a greater methamphetamine problem due to the lack of regular surveying and investigation by the police or other relevant government officers. Females are less likely than males to be investigated by the police. Youths who are living in villages located closer to the border area, particularly Thailand-Myanmar, have a greater problem with methamphetamine use than youths living in a village located closer to a large city. Villages located closer to border areas have a higher feasibility of accessing drugs than do villages near a large city.

A 21-year-old Lahu man said the following:

*“I live and grew up here, very far from the city. No police and the village headman does not care if the drug (methamphetamine) problem goes on. The drug is very easy to get in this village. You just ask someone here, and you will get it immediately, particularly women.”* (Lahu male youth, 21 years old, # 2).

He added the following:

*“Do you know that when the police come here, they always look for men and investigate them, but they ignore women. Then, women say that they are safe because the police do not suspect them.”* (Lahu male youth, 21 years old, # 2).

*He added the following:*

*“One night 4 years ago, I joined a party with my friends in the village. We had a new year’s ceremony. Sometime after the party, one of my friends gave me a pill and said that it could make me happy. I took it. You know, everyone who joined the party used it eventually.”* (Lahu male youth, 21 years old, # 2).

### Five supportive factors in the initiation of methamphetamine use

Supportive factors were defined as the individual perceived factors to support the initiation of methamphetamine use among the Akah and Lahu youths. There were five factors found in the analysis.

#### Social norm perceptions

All males and more than half of the females reported that methamphetamine use was common in Akha and Lahu villages. A few young people did not use methamphetamine. People used it for different reasons; some used it while working on the farm, but some used it at night while meeting with their friends. The use of methamphetamine in the Lahu villages, particularly in remote areas and among males of working age, was also very common.

A Lahu man said, *“80% of males and 60% of females in my village use methamphetamine.”* (Lahu male youth, 19 years old, # 01).

This statement was supported by another Akha man:

*“I think more than 80% of my friends use the drug (methamphetamine), but I am not sure about females … maybe less than 40%. Most women use it while drinking alcohol.*” (Akha male youth, 21 years old, # 04).

He added the following:

“*I have used it for 2 years. My reason for using it the first time was just funny and I did not think it was wrong; everyone used it. In our village, police do not conduct checks.”* (Akha male youth, 21 years old, # 04).

#### School dropout

A large proportion of Akha and Lahu youths who were exposed to methamphetamine were people out of school. There were several reasons for leaving school, such a lack of family support and poor academic performance. Commonly, youths who were out of the school had a greater probability of methamphetamine use than those who were attending school due to having a greater chance of exposure to people who were in using methamphetamine and living in the same village. Ultimately, they would be motivated to initiate methamphetamine use by their peers through several activities, such as a new year’s party or after a sports game in the evening. A longer period of exposure to people who were using drugs in the village increased the odds of methamphetamine use initiation among the Akha and Lahu youths. Those who were unemployed and blamed by their parents were at much a greater risk of initiating methamphetamine use.

A 19-year-old Lahu man said the following:

*“I left school for 3 years and have not worked since then. I just stay at home and drink with friends in the evening. I feel bored and hate myself. I spend most of my daytime with my friends and drinking alcohol. A couple years ago, I started using the drug (methamphetamine).”* (Lahu male youth, 19 years old, # 04).

A 16-year-old Lahu woman said the following:

*“I left school when I was 12. I got very poor grades, and then I decided not to continue my studies. A couple years ago, when we had a new year’s ceremony, I started to use the drug (methamphetamine). I know it is not good, but it makes me happy and helps me make friends.”* (Lahu female youth, 16 years old, # 19).

#### Guardianship and parenting

There were several problems at the family level that affected the initiation of methamphetamine use among the Akha and Lahu youths, such as not having parents, parental divorce, family conflicts, and poor parenting. More than half of the participants were orphans. Four Akha participants and 7 Lahu participants reported that their parents were imprisoned for illegal drug cases at the time of the interview. Another 5 Akha participants and 4 Lahu participants reported that at least one of their parents had died or that their parents did not live together (divorced). Family relationships and parenting styles were found to be the perceived factors influencing the initiation of methamphetamine use among the Akha and Lahu youths.

A 16-year-old Akha man said the following:

*“I have no mother and no father at home. Both of them were imprisoned for drug-related offenses 7 years ago. I live with one younger sister and one younger brother. We are three people living together. In addition, every day, my grandmother gives us food. I have to work outside, and the employees do not want me to work with them. They said I was too young with no energy to work in the farm. Then, I started to use the drug (methamphetamine) and showed them that I could do it. I need to survive and I cry almost every night while my sister and brother go to bed. I have no choice!”* (Akha male youth, 16 years old, #10).

A 21-year-old Akha woman said the following:

*“Today, I am living with my mother and father-in-law. My father died ten years ago in a traffic accident. I have three younger brothers. I wake up early in the morning to prepare food and everything for all of them. My parents were kind when they did not drink alcohol; however, they caused trouble in the family whenever they had alcohol. I was unhappy and one day I met my old friend who came back from work in the city. She invited me to use the drug (methamphetamine), and I have been using it ever since then.”* (Akha female youth, 21 years old, #19).

A 15-year-old Lahu man said the following:

*“I am now living with my parents and two younger brothers. My parents work as daily wage employees on a Thai farm. They get 300 baht per day per person. Most evenings, both of my parents drank alcohol with their coworkers and haggled in small to serious conflicts. One day my mother’s head was broken because it was bashed by my father. I really know that my brothers and I are living in an uncomfortable family, particularly in the evening. We go to bed early every single day to avoid trouble. I had started to use the drug (methamphetamine) a couple years ago.”* (Lahu male youth, 15 years old, # 06).

#### Poor economic status

Individual income and the family’s poor economic status were also detected as the individual perceived factors influencing the initiation of methamphetamine use among the Akha and Lahu youths. Families with poor economic status could not support their children to have good opportunities in their lives, including schooling. The children needed to drop out of school and work in the field to support the family while they were young. When a family had poor economic status, children became a person who used methamphetamine for several reasons, such as improving their energy for work, relieving pain, adjusting their social relationships with their peers and addressing mental problems.

A 15-year-old Lahu man said the following:

“*I just left school. I was shy around my friends because my parents did not give me money for snacks before going to school. We are poor. I had no food for breakfast after waking up, and I did not know where my parents were. I did not see them and there was no food. Then, I decided not to go to school*.” (Lahu male youth, 15 years old, # 02).

A 17-year-old Lahu woman said the following:

*“I am living with my very poor family. My parents have five children. I am definitely the oldest child. They worked as daily wage laborers, with 250 baht/day for my mother and 300 baht/day for my father. Both of them use the drug (methamphetamine). I had to leave school due to no support from my parents. I help them work on the farm, and sometimes I get money. One evening, I came back from work with my friends; they asked me to drink alcohol, and later on, we initiated drug (methamphetamine) use.”* (Lahu female youth, 17 years old, #16).

A 19-year-old Akha man said the following:

*“I have no choice in my life. I did not get any support from my parents to go to school. They told me that they did not have money to give me to go to school; I understood. I felt heartbroken and cried many times and asked myself why it happened to me. Why did I not have rich parents so I could do what I wanted? I always knew that drug (methamphetamine) use was not a good thing, but when I feel heartbroken, only a drug can help me relieve the pain*. *However, I hope that I will find a better way in the future.”* (Akha male youth, 19 years old, #01).

A 23-year-old Akha woman said the following:

*“I live with my poor family, which consists of 12 persons. I am the first child of my parents and graduated primary school in the village. I know that my parents work hard every day to support their children. I decided to work in Bangkok with the hope of taking some money back to support my family. Finally, I worked as a sex worker with no better option in Bangkok. Actually, I did not want to be a sex worker, but I had no choice. I started using methamphetamine to relieve the discomfort and pain from work and health problems. Last year, in March 2017, I got a severe illness and tested positive for HIV. I just got back to the village a couple of months ago.”* (Akha female youth, 23 years old, #15).

#### Not having Thai citizenship

Having a Thai national Identification Card (ID), which is used to identify Thai citizens, is also the individual perceived factors influencing methamphetamine use among the Akha and Lahu youths. The hill tribes who have Thai citizenship and a Thai national ID card have full rights to move and work in large cities. Those people who work in a large city have a greater chance of earning money and improving the quality of their family members’ lives, including increasing the affordability of their children’s education. Many young hill tribe people, including Akha and Lahu youths, want to have a Thai ID card. This card represents a better chance of getting a better job and earning a better income. Conversely, those who do not have a Thai ID card are easily engaged in selling and using methamphetamine due to having no other options to work for a better income to support their family. Poor economic status leads to stress and, ultimately, methamphetamine use.

A 16-year-old Lahu woman said the following:

*“I do not know what happened. I was born in Thailand, but I did not get a Thai ID card. Many of my friends are working in Chiang Mai and Bangkok, and they always send back money to their families. I want to do that. Currently, I cannot even study in a school or work in a big city because I have no Thai ID card. It makes me feel bad.”* (Lahu female youth, 16 years, # 15).

She added the following:

*“I think it is not too fair to me. I asked the government officers to get a Thai ID card, but I always get a negative response from them. They told me that I have to find some people to be witnesses because my parents died many years ago. Yes, if you still live in this village, you would use the drug (methamphetamine). So, I do!”* (Lahu female youth, 16 years, #15).

A 20-year-old Akha man said:

*“Today, I have no Thai ID card even though I was born here in this village. I have lived here for almost 20 years. Everyone in the village knows me, but I do not know why I did not get a Thai ID card. Before leaving school many years ago, I planned to work in Chiang Mai to earn money. Unfortunately, on the way to Chiang Mai, I was caught by a policeman, and all my dreams were broken. I have nothing now. Yes, I feel very bad in my life and started to use the drug three years ago.”* (Akha male youth, 20 years old, # 5).

## Discussion

This qualitative approach explained that four major perceived factors (low self-esteem, family member use, positive expectation regarding methamphetamine use, and availability), and five supportive factors (social norm perception, school dropout, family level problems, poor economic, and no Thai citizenship) were found to contribute to the initiation of methamphetamine use among the Akha and Lahu youths in northern Thailand in the hill tribe villages. Low self-esteem, methamphetamine use in the family, positive expectations of methamphetamine use, and availability were the major perceived factors to the initiation of methamphetamine use. Social norm perceptions, school dropout, family-level problems, poor economic status, and not having a Thai citizenship card were supportive factors to the initiation of methamphetamine use among the hill tribe Akha and Lahu youths in northern Thailand.

A study in rural Chiang Mai Province, Thailand, among young adults reported that methamphetamine use was associated with high levels of depressive symptoms and low self-esteem personality characteristics [16]. Garofalo et al. [17] also reported that low self-esteem personality characteristics were correlated with methamphetamine use among young men in Chicago, United States.

Lynne et al. [18] demonstrated that parents’ use of methamphetamine during children’s early stages of life and throughout childhood was associated with the initiation of methamphetamine use among youths aged 15–24 years. Russell et al. [19] also reported that family use of methamphetamine was one of the significant factors contributing to the initiation of amphetamine use in youth. These findings supported our finding that Akha and Lahu youths who live in a family in which parents use methamphetamine were more likely to use methamphetamine at some point in their lives.

Noroozi et al. [20] also reported that some outcome expectancies, such as increased energy to improve occupational functions and tackle fatigue, were reported as contributing factors in the initiation of methamphetamine use among the youth in Tehran. Moreover, Abelman [21] reported that students aged 18–25 years from several countries had used methamphetamine because they expected better scores on their examinations. These findings support those of our study indicating that a positive expectation of methamphetamine use, particularly increased energy for work, was a contributing factor to the initiation of methamphetamine use among the Akah and Lahu hill tribe youths in northern Thailand.

A qualitative study of the initiation of methamphetamine use among Thai youths reported that availability was a major factor contributing to their initiation of methamphetamine use [22]. A systematic review conducted by Russell et al. [19] examining the risk factors for methamphetamine use in youth reported that availability was one of the major factors contributing to the initiation of methamphetamine use among youth. A study in Tehran reported that availability was an important contributing factor to the initiation of methamphetamine use among youths [23]. These findings coincide with those of our study indicating that the availability of methamphetamine, particularly in the Akha and Lahu villages, was one of the major contributing factors to the initiation of methamphetamine use among youths.

The social norm of the perception of methamphetamine use was one of the supportive factors in the initiation of methamphetamine use among the Akha and Lahu hill tribes in northern Thailand. This finding is supported by the study of Barman-Adhikari et al. [24], who reported that social norms, particularly in methamphetamine use, were influenced by social networks and their peers among homeless youths in California, United States. Moreover, Brian et al. [23] reported that social norm perceptions in the use of methamphetamine were a significant factor in initiating use among Chinese youths who used methamphetamine. A qualitative study of the factors influencing the use of methamphetamine among youths in Tehran showed that social acceptance was one of the perceived factors influencing the initiation of methamphetamine use [20].

A cluster randomized trial study was conducted in Thailand and found that youths who were not fully attending school were at risk of initiation of methamphetamine use [25]. A study in the United States also reported that children who were school dropouts were more likely to initiate amphetamine use than those who were in school [26]. These results supported our finding that youths who are outside school have a greater chance of initiating methamphetamine use than those who are still in school.

Hemovich et al. [26] also that family structure and family problems were highly influential factors of the initiation of drugs, particularly methamphetamine, in the United States. A study of family function and substance use among Hispanic and Anglo runaway youths also found that family problems were detected as the initiating factor of drug use in youths [27]. Russell et al. [19] performed a systematic review on risk factors for methamphetamine use in youth and found that family problems were significantly associated with the initiation of methamphetamine use in youths. Noroozi et al. [20] also reported that poor parenting and family problems were factors contributing to the initiation of methamphetamine use among youths in Tehran. These reports support our finding that family problems were a supportive contribution to the initiation of methamphetamine use among the Akah and Lahu youths in northern Thailand.

In our study, we found that poor economic status and low levels of education due to dropping out of school were supportive perceived factors influencing methamphetamine use among Akah and Lahu youths in Thailand. Several studies have also reported that youths who lived in remote areas, had low levels of education and had poor families were at risk of the initiation of methamphetamine use [28–32]. Ponicki et al. [33] also reported that even in the United States, youths who lived in poor families were more likely to initiate amphetamine use than those living in high-income families. A study in Cape Town, South Africa, found that poor family economic status was one of the most significant contributions to the initiation of methamphetamine use among youths [34].

The issue of not having Thai citizenship was found to be one of the supportive perceived factors influencing the initiation of methamphetamine use among the Akha and Lahu hill tribe youths in Thailand. It might be anyone who has a Thai citizenship card will be allowed to pursue a university degree and work in a professional job sector. In contrast, those who do not have a Thai citizenship card are not allowed to pursue university study and work in a professional work sector [35–37]. With these complicated situations, youths become vulnerable to methamphetamine use at an early age. Many of them decide to engage in commercial sex work in a large city. Whether male or female, those who engage in sex work are more likely to initiate methamphetamine use. Mostafa et al. [38] reported that young women who engage in commercial sex work were at risk of methamphetamine use, particularly in poor settings. Moreover, Sin et al. [39] reported that engaging in male sex work in Malaysia was a motivating factor in the initiation of methamphetamine use. Several other studies [40–42] have reported that young men who engaged in commercial sex work in a large city tended to initiate methamphetamine use.

## Conclusions

Akha and Lahu hill tribe youths are vulnerable to the initiation of methamphetamine use due to five supportive perceived factors (social norm perceptions, school dropout, family-level problems, poor economic status, and lack of Thai citizenship) and four major perceived factors (low self-esteem, use by family members, positive expectations of methamphetamine use, and availability). Today, both Akha and Lahu youths are faced with different challenges from childhood to adulthood. As a result, some youths become individuals who used methamphetamine. Low economic status and low educational levels are the major factors influencing the initiation of methamphetamine use among Akha and Lahu youths. They do not have many choices with respect to jobs due to their poor education and lack of a Thai ID card. As a result, some youths decided to engage in commercial sex. These populations need help in transitioning through the most vulnerable stage for the initiation of methamphetamine use, i.e., from childhood to adulthood. Government and nongovernmental agencies with a mission and expertise in addressing drug problems should collaborate and integrate their programs to develop effective and sustainable ways to protect these youths from methamphetamine use. Improving access to education for Akha and Lahu youths to improve their chances of working in professional job sectors would be the best option to reduce the problem of the initiation of methamphetamine use in these populations. Public health interventions that focus on sharing knowledge of the negative effects of methamphetamine use among both male and female youths should be immediately considered. Finally, integrative development and implementation among several agencies, including public health professionals, psychologists, and the police, in addressing the circumstances of the hill tribe villages by focusing on youths should be an area of serious focus.

## Additional file

Additional file 1: A sixteen-question guideline. (DOCX 13 kb)

## Data Availability

There is no available on raw data.

## Abbreviation

ID: Identification card
