# Supplementary material for: Perceived factors influencing the initiation of methamphetamine use among Akha and Lahu youths: a qualitative approach
Source: BMC Public Health. 2019 Jun 28;19:847. doi: 10.1186/s12889-019-7226-y (PMC6599247; doi:10.1186/s12889-019-7226-y)
Supplement: Supplementary file 1 — A sixteen-question guideline. (DOCX 13 kb) [file 12889_2019_7226_MOESM1_ESM.docx]

**A sixteen-question guideline**

1. How long you have lived in Thailand and this village? Do you have Thai ID card (which is 13 digits)?

2. Could you please tell me about your family? Do your parents live together? How many people live together?

3. How about the relationship of your parents and your family member?

4. Could you please tell me detail while you were young?

5. Did you have any conflict with your parents or your family member? Is it serious?

6. Did you have any dream while you were young? Is it getting real? Why?

7. Could you please tell me your study history? Did you have any problems regarding your study? Why you did not attend school?

8. Is it important to study? Why?

9. Do/did you have any serious problem in your life? When and how did you address it?

10. What do you think about methamphetamine? Is it impact to your life? Your family? Your friends? Your village?

11. Do you use methamphetamine? If yes, how often?

12. Could you please tell me on reasons in your first use of amphetamine? How did you get it?

13. Did you have any bad experience regarding use methamphetamine?

14. Do you have any friends who use amphetamine? Who lives in this village and outside the village?

15. Why people use methamphetamine?

16. What do you think on the role of community leaders to protect and cope amphetamine problem in village?
